# Molecular identification and antifungal susceptibility testing of *Aspergillus* species among patients with chronic pulmonary aspergillosis in Nigeria

**DOI:** 10.4102/ajlm.v14i1.2674

**Published:** 2025-07-11

**Authors:** Adeyinka A. Davies, Bram Spruijtenburg, Eelco F.J. Meijer, Iriagbonse I. Osaigbovo, Oluwaseyi Balogun, Abiola Adekoya, Titilola Gbaja-Biamila, Jacques F. Meis, Rita Oladele

**Affiliations:** 1 Department of Medical Microbiology and Parasitology, Olabisi Onabanjo University Teaching Hospital, Sagamu, Nigeria; 2 Department of Medical Microbiology and Immunology, Canisius-Wilhelmina Hospital (CWZ)/Dicoon, Nijmegen, the Netherlands; 3 Radboudumc-CWZ Center of Expertise for Mycology, Nijmegen, the Netherlands; 4 Department of Medical Microbiology, School of Medicine, University of Benin, Benin City, Nigeria; 5 Department of Biomedical Engineering, University of Lagos, Idi-Araba, Surulere, Nigeria; 6 Department of Radiology, Olabisi Onabanjo University Teaching Hospital, Sagamu, Nigeria; 7 Nigeria Institute of Medical Research, Yaba, Lagos, Nigeria; 8 Saint Louis University College of Public Health and Social Justice, St. Louis, Missouri, United States of America; 9 Institute of Translational Research, Cologne Excellence Cluster on Cellular Stress Responses in Aging-Associated Diseases (CECAD) and Excellence center for Medical Mycology, University of Cologne, Cologne, Germany; 10 Department of Medical Microbiology and Parasitology, Lagos University Teaching Hospital, Idi-Araba, Lagos, Nigeria

**Keywords:** chronic pulmonary aspergillosis, antifungal susceptibility test, phylogenetic analysis, tuberculosis, *Aspergillus* spp., Nigeria

## Abstract

**Background:**

Triazole resistance in *Aspergillus* spp. has therapeutic implications for managing chronic pulmonary aspergillosis (CPA) worldwide. However, antifungal susceptibility testing (AFST) is not routinely performed in Nigeria, a country with a high CPA burden.

**Objective:**

This study aimed to confirm the identity of *Aspergillus* spp. isolated from patients with CPA using molecular methods, determine their antifungal susceptibility profile, and ascertain phylogenetic relatedness.

**Methods:**

This study examined 47 *Aspergillus* isolates from sputum samples obtained in a prospective longitudinal study of CPA prevalence among 141 consenting symptomatic tuberculosis patients in Lagos, Nigeria, between June 2021 and May 2022. The preliminary phenotypically identified *Aspergillus* spp. were further identified by amplifying the calmodulin gene and performing AFST against seven antifungal agents using the Clinical Laboratory Standard Institute (CLSI) micro-dilution method, as well as determining their phylogenetic relatedness.

**Results:**

The 51 patients who met the diagnostic criteria for CPA included 30 (59.0%) male and 21 (41.0%) female patients (age range: 17–68 years). Thirty-six (71.0%) had positive *Aspergillus* cultures. An isolate, initially identified phenotypically as *A. fumigatus*, was reidentified as *A. pseudonomiae*. Phylogenetic analysis on *A. fumigatus* and *A. flavus* isolates suggested the absence of clonal transmission. All isolates were susceptible to the tested antifungals.

**Conclusion:**

Clinical *Aspergillus* isolates from azole-naïve patients with CPA did not demonstrate triazole resistance. Nonetheless, AFST is required for patients on long-term azole therapy and systematic surveillance of clinical and environmental isolates is recommended to detect the emergence of azole-resistant phenotypes.

**What this study adds:**

This study underscores the importance of routine surveillance for antifungal resistance to detect the occurrence of resistance strains early in clinical settings, as this has therapeutic implications for patients harbouring resistant phenotypes.

## Introduction

In 2022, the World Health Organization released the first fungal priority pathogens list, with *Aspergillus fumigatus* categorised as a critical pathogen.^1^ *Aspergillus fumigatus* accounts for many allergic and invasive lung conditions, principal amongst those with chronic pulmonary aspergillosis (CPA). Outside Europe, specifically in Asia and Africa, non-*fumigatus* species such as *A. flavus* and *A. terreus* are also increasingly implicated.^2,3,4^ Regardless of the *Aspergillus* spp., CPA is a slowly progressing but debilitating disease which affects individuals with structural lung defects. The annual incidence is 1 837 272 cases, of which about 340 000 (18.5%) die.^5^ The prevalence of CPA in tuberculosis-treated patients has been reported as 49.7% in Nigeria,^6^ 50% in Ghana,^3^ and 57% in India.^7^ A combination of symptomatology persisting over 3 months, radiological findings, positive cultures, and serological tests is required to diagnose. The disease often requires long-term treatment with systemic antifungal agents, preferably triazoles such as itraconazole or voriconazole administered orally. Antifungal therapy improves symptoms and reduces morbidity and mortality of the disease in most patients. Identifying clinical and microbiological resistance during treatment is of utmost importance to limit relapse and improve the long-term survival rate.

Besides being useful for treating all forms of aspergillosis,^8^ triazoles, which have a broad spectrum of antifungal activity, are also used as environmental fungicides to prevent fungal spoilage of crops by phytopathogens.^9^ Cross-resistance between medical and environmental triazoles has been documented.^10^ Azole resistance in *A. fumigatus* was first reported in clinical isolates of azole-experienced CPA patients in 1997^11^ and azole-naïve invasive aspergillosis patients in 2014.^12^ Since then, several countries have reported resistance to triazoles in clinical and environmental isolates,^8,13^ including Africa, where documented resistance to environmental *Aspergillus* isolates has been reported from the Eastern and Western regions.^14^ Also, TR34/L98H was described from a clinical *Aspergillus* isolate in Kenya.^15^ This emphasised that azole resistance in *Aspergillus* spp. is present, although the lack of proficiency and resources to routinely perform antifungal susceptibility testing (AFST), underestimation of azole resistance in *A. fumigatus* prevalence persists. However, the rising trends in azole-resistant *A. fumigatus* globally made the panel of experts recommend subjecting all clinical *Aspergillus* spp. isolates to AFST,^16^ which has not been feasible in Africa. Additionally, surveillance data on triazole resistance in clinical *Aspergillus* spp. from Africa has been sparse. Therefore, this study aimed to determine the antifungal susceptibility profile of clinical isolates of *Aspergillus* after identifying them to the species level using molecular methods.

## Methods

### Ethical considerations

This study was approved by the Institutional Ethical Committees of Lagos University Teaching Hospital in December 2020 (reference number: ADM/DCST/HREC/APP/3868) and by the Nigeria Institute of Medical Research in January 2021 (reference number: IRB/20/096). Written informed consent was obtained from all participants, with data entered into a private, secure computer with access limited to investigators. Test results were kept confidential and disclosed only to the patient and their healthcare provider.

### Study design

This was a descriptive study of *Aspergillus* isolates obtained from a previously reported prospective longitudinal study conducted to determine the CPA incidence in two tuberculosis treatment clinics in Lagos, Nigeria (Lagos University Teaching Hospital and Nigeria Institute of Medical Research, Yaba), between 01 June 2021 and 31 May 2022.^6^ In the previous study, 141 consenting adult patients who were previously treated for tuberculosis (1–4 years earlier) and were clinically classified as retreatment (*n* = 79; 56.0%) and post-tuberculosis treatment (*n* = 62; 44.0%) patients were recruited. Fifty-one patients were diagnosed with CPA based on a combination of positive Bordier *Aspergillus*-specific IgG assays (Bordier Affinity Products, Crissier, Switzerland) in patient serum, typical chest X-ray features of CPA, and isolation of *Aspergillus* spp. from high-volume sputum cultures,^17^ of these, 47 *Aspergillus* spp. were cultured from the sputum of 36 patients. The demographic characteristic and *Aspergillus* spp. distributions were analysed with IBM Statistical Package for Social Science software version 25 (IBM Corp., Armonk, New York, United States).

#### Isolate culture and DNA extraction

As part of the current study, phenotypic identification of isolates recovered from sputum was carried out at the Mycology Laboratory of Lagos University Teaching Hospital. Identification was based on colonial morphology and microscopic features as previously described by Davies et al.^6^ Thereafter, isolates were transported to Canisius-Wilhelmina Ziekenhuis Hospital (Nijmegen, the Netherlands) on sterile filter paper impregnated with Tween 20 for molecular identification, performed between 04 December 2022 and 15 February 2023.

At Canisius-Wilhelmina Ziekenhuis Hospital, the isolates were stored at -80 °C, according to standard procedures, until use. The isolates were grown on Sabouraud dextrose agar plates (Oxoid, Hampshire, United Kingdom) for 7 days at 30 °C. For the DNA extraction, isolates were resuspended in 400 µL MagNA Pure bacteria lysis buffer (Roche Diagnostics GmbH, Mannheim, Germany) and MagNA Lyser green beads (Roche Diagnostics GmbH, Mannheim, Germany).^18^ The conidia were lysed by a MagNA Lyser instrument (Roche Diagnostics GmbH, Mannheim, Germany) for 30 s at 6500 rpm. All tubes were consecutively inactivated at 100 °C for 10 min. DNA was extracted and purified using the MagNA Lyser system (Roche Diagnostics GmbH, Mannheim, Germany). The MagNA Pure and Vial NA Small Volume Kit was used following the manufacturer’s instructions, as previously described by De Groot et al.^19^

#### Molecular species identification

As part of the current study, a polymerase chain reaction amplifying the calmodulin (*CaM*) gene was used for species identification using primers Cmd5 5’-CCGAGTACAAGGARGCCTTC-3’ and Cmd6 5’-CCGATRGAGGTCATRACGTGG-3’, as previously described.^20^ Generated amplicons were purified according to the Ampliclean protocol (NimaGen, Nijmegen, the Netherlands) and the sequencing polymerase chain reaction was performed using 0.5 µL BrilliantDye premix, 1.75 µL BrilliantDye 5x sequencing buffer (NimaGen, Nijmegen, the Netherlands), 1 µL Cmd6 primer (5.0 µM), 5.75 µL water and 1 µL purified DNA. Ensuing amplicons were purified using the D-Pure purification method (NimaGen, Nijmegen, the Netherlands) and sequenced on a 3500 XL genetic analyzer (Applied Biosystems, Foster City, California, United States). Calmodulin sequences of *A. fumigatus* CBS 487.65 (AB259965.1), *A. niger* CBS 101700 (GU195633.1), *A. flavus* CBS 117622 (EF202068.1), and *A. pseudonomiae* DTO:267-I4 (KP330066.1) were extracted from the National Center for Biotechnology nucleotide database. Alignment and tree building were done with the Clustal Omega multiple alignment algorithm.^21^ Visualisation and editing were made with iTOL v6 (https://itol.embl.de).^22^ Calmodulin sequences generated in the present study were deposited under Genbank accession numbers OQ915062–OQ915108.

#### Antifungal susceptibility testing

As part of the current study, AFST against amphotericin B (Bristol Myers Squib, Woerden, the Netherlands), itraconazole (Janssen Cilag, Beerse, Belgium), voriconazole (Pfizer Central Research, Sandwich, United Kingdom), posaconazole (Merck, Haarlem, the Netherlands), isavuconazole (Basilea Pharmaceutica, Basel, Switzerland), anidulafungin (Merck, Haarlem, the Netherlands) and micafungin (Merck, Haarlem, the Netherlands) was performed by broth microdilution following the Clinical Laboratory Standard Institute (CLSI) M38-A2 protocol.^23^ The spores were diluted in RPMI 1640 medium and adjusted with a Genesys 20 spectrophotometer (Thermo Fisher Scientific, Waltham, Massachusetts, United States)^19^ to obtain final concentrations of 1 × 10^5^ to 5 × 10^5^ CFU/mL suspension and subsequently transferred into microtiter plates with the appropriate antifungal concentrations. The plates were incubated at 35 °C for 48 h and visually interpreted. Minimal inhibitory concentrations (MICs) were read as the lowest antifungal concentration with a 100% growth reduction when compared to the growth control.^18^ For the echinocandins, minimal effective concentrations were determined microscopically as the lowest drug concentration with the growth of small, rounded, compact hyphal forms compared to the hyphal growth in the control. Tentative antifungal breakpoints based on expert opinion and epidemiological cutoff values were implemented for different *Aspergillus* spp. According to CLSI, the epidemiological cutoff value for *A. fumigatus* is 2 µg/mL for testing amphotericin B, in comparison, 4 µg/mL for *A. flavus*, whereas, for voriconazole, ≤ 0.5 µg/mL was interpreted as susceptible, 1 µg/mL as intermediate, and ≥ 2 µg/mL as resistant.^24^

#### Short tandem repeat genotyping

As part of the current study, *A. fumigatus* and *A. flavus,* isolates were genotyped as previously described by De Valk et al.^25^ and Rudramurthy et al.^26^ In short, multiplex polymerase chain reaction reactions amplifying nine short tandem repeat markers were performed using 1× FastStart *Taq* polymerase buffer without MgCl_2_, deoxynucleoside triphosphates (0.2 mM), MgCl_2_ (3 mM), forward and reverse primers (5 µM), 1 U FastStart *Taq* polymerase (Roche Diagnostics GmbH, Mannheim, Germany), and isolated DNA on a thermocycler (Biometra, Westburg, Göttingen, Germany).^18^ Products were analysed on a 3500 XL genetic analyser (Applied Biosystems, Foster City, California, United States), and corresponding copy numbers were determined and analysed using GeneMapper software (Applied Biosystems, Foster City, California, United States), as previously described by Spruijtenburg et al.^27^

### Data analysis

The demographic data were entered directly into Microsoft Excel (Microsoft, Redmond, Washington, United States), while the laboratory results were first entered into a book dedicated solely to this study and later transferred into Microsoft Excel. The participants were categorised into re-treatment and post-tuberculosis treatment groups. Descriptive analysis was done with IBM Statistical Package for Social Science software version 25 (IBM Corp., Armonk, New York, United States), with data presented in frequency and percentages. These data were then displayed in tabular form. A multistate categorical coefficient was used to determine genetic relatedness between isolates (BioNumerics v7.6.1 software; Applied Mathd NV, Sint-Martens-Latem, Belgium).

## Results

Of the 51 patients who met the case definition for CPA, 47 *Aspergillus* spp. were recovered from the sputum cultures of 36 (70.6%) patients. Participants included 19 (37.3%) male patients and 17 (33.3%) female patients, with ages ranging from 21 to 75 years. *Aspergillus flavus* (*n* = 18; 35.3%) was the most common species isolated. However, multiple *Aspergillus* spp. were also isolated from 10 patients, with nine consisting of a combination of two different species, while the remaining patient had a mixture of *A. flavus, A. fumigatus,* and *A. niger* isolated (Table 1).

**TABLE 1:** Demographic, clinical characteristics, and culture findings of patients recruited from Lagos University Teaching Hospital and Nigeria Institute of Medical Research, Yaba, Nigeria, between 01 June 2021 and 31 May 2022.

Participant characteristics	*n*	%	Range
**CPA participants**	51	100.0	-
Age	-	-	17–68
Sex	-	-	-
Male	30	59.0	-
Female	21	41.0	-
**Positive sputum culture**	36	70.6	-
Age	-	-	21–75
Sex	-	-	-
Male	19	37.3	-
Female	17	33.3	-
**Treatment states**			
Retreatment tuberculosis	22	43.1	-
Post-tuberculosis	14	27.5	-
***Aspergillus* species**			
*A. flavus*	18	35.3	-
*A. niger*	2	3.9	-
*A. fumigatus*	5	9.8	-
*A. pseudonomiae*	1	2.0	-
*A. flavus* and *A. fumigatus*	5	9.8	-
*A. flavus* and *A. niger*	4	7.8	-
*A. niger, A. fumigatus,* and *A. flavus*	1	2.0	-

CPA, chronic pulmonary aspergillosis.

### Molecular identification

**FIGURE 1:**
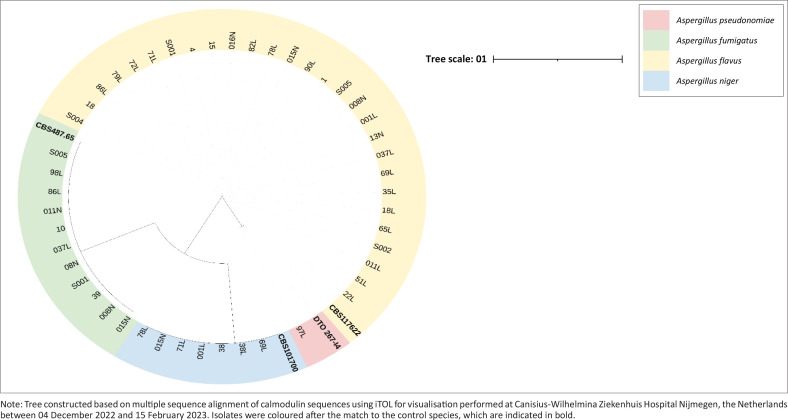
Phylogenetic tree of *Aspergillus* species from Nigeria, between 01 June 2021 and 31 May 2022.

Genotypic species determination was based on calmodulin sequencing using Clustal Omega multiple sequence alignment, which resulted in the identification of 28 *A. flavus sensu stricto* strains, 11 *A. fumigatus sensu stricto* strains, seven *A. niger sensu stricto* strains, and one *A. pseudonomiae* (Figure 1, Table 2).

**TABLE 2:** Isolate overview of *Aspergillus* species, including minimal inhibitory concentrations in mg/L and host clinical information, all isolated from sputum. Analysis was performed at Canisius-Wilhelmina Ziekenhuis Hospital Nijmegen, the Netherlands between 04 December 2022 and 15 February 2023.

Patient ID	Sex	Age (years)	Treatment	*Aspergillus* species	Minimum inhibitory concentration of tested antifungal drugs (mg/L)						
					Amphotericin B	Itraconazole	Voriconazole	Posaconazole	Isavuconazole	Anidulafungin	Micafungin
001L	M	48	Retreatment TB	*A. niger*	0.063	0.500	1.000	0.125	1.000	≤ 0.008	≤ 0.008
				*A. flavus*	0.250	0.063	0.500	0.063	0.500	≤ 0.008	≤ 0.008
010	M	28	Post TB	*A. fumigatus*	0.250	0.250	0.500	0.031	0.500	≤ 0.008	≤ 0.008
011L	F	22	Post TB	*A. flavus*	0.250	0.063	0.250	0.063	0.250	≤ 0.008	≤ 0.008
18L	F	36	Retreatment TB	*A. flavus*	0.500	0.053	0.500	0.063	0.500	≤ 0.008	≤ 0.008
15	M	52	Post TB	*A. flavus*	0.500	0.063	0.500	0.125	0.500	≤ 0.008	≤ 0.008
22L	M	59	Retreatment TB	*A. flavus*	0.250	0.125	0.500	0.063	0.500	≤ 0.008	≤ 0.008
38	M	50	Retreatment TB	*A. niger*	0.063	0.250	0.500	0.063	1.000	≤ 0.008	≤ 0.008
35L	F	62	Retreatment TB	*A. flavus*	0.250	0.063	0.250	0.031	0.500	≤ 0.008	≤ 0.008
037L	M	38	Retreatment TB	*A. fumigatus*	0.250	0.125	1.000	0.031	1.000	≤ 0.008	≤ 0.008
				*A. flavus*	0.500	0.063	0.500	0.063	0.500	≤ 0.008	≤ 0.008
38L	F	28	Post TB	*A. niger*	0.125	0.250	1.000	0.125	1.000	≤ 0.008	≤ 0.008
39	M	28	Retreatment TB	*A. fumigatus*	0.250	0.125	0.500	0.031	0.500	≤ 0.008	≤ 0.008
51L	M	22	Retreatment TB	*A. flavus*	0.500	0.063	0.250	0.063	0.500	≤ 0.008	≤ 0.008
65L	M	35	Post TB	*A. flavus*	0.500	0.125	0.500	0.063	0.500	≤ 0.008	≤ 0.008
69L	F	33	Retreatment TB	*A. niger*	0.063	0.063	0.500	0.063	0.500	≤ 0.008	≤ 0.008
				*A. flavus*	0.063	0.063	0.500	0.063	0.500	≤ 0.008	≤ 0.008
71L	F	40	Retreatment TB	*A. niger*	0.125	0.250	0.500	0.063	0.250	≤ 0.008	≤ 0.008
				*A. flavus*	0.500	0.063	0.250	0.031	0.063	≤ 0.008	≤ 0.008
72L	F	75	Retreatment TB	*A. flavus*	0.250	0.125	0.500	0.031	0.500	≤ 0.008	≤ 0.008
78L	F	21	Post TB	*A. niger*	0.125	0.250	0.500	0.063	0.500	≤ 0.008	≤ 0.008
				*A. flavus*	0.500	0.063	0.500	0.031	0.500	≤ 0.008	≤ 0.008
79L	F	62	Retreatment TB	*A. flavus*	1.000	0.125	1.000	0.031	0.500	≤ 0.008	≤ 0.008
82L	M	23	Post TB	*A. flavus*	1.000	0.125	0.500	0.063	0.500	≤ 0.008	≤ 0.008
86L	M	25	Post TB	*A. fumigatus*	0.250	0.125	1.000	0.063	1.000	≤ 0.008	≤ 0.008
				*A. flavus*	1.000	0.063	1.000	0.063	0.500	≤ 0.008	≤ 0.008
90L	F	30	Post TB	*A. flavus*	0.250	0.063	0.500	0.031	0.500	≤ 0.008	≤ 0.008
97L	M	50	Retreatment TB	*A. pseudonomiae*	0.500	0.250	1.000	0.125	1.000	≤ 0.008	≤ 0.008
98L	M	31	Retreatment TB	*A. fumigatus*	0.250	0.063	0.250	0.031	0.250	≤ 0.008	≤ 0.008
S001	F	34	Post TB	*A. fumigatus*	0.250	0.125	0.500	0.063	0.500	≤ 0.008	≤ 0.008
				*A. flavus*	0.500	0.063	0.500	0.063	0.500	≤ 0.008	≤ 0.008
S002	F	58	Post TB	*A. flavus*	0.500	0.125	0.500	0.063	0.500	≤ 0.008	≤ 0.008
S004	M	38	Post TB	*A. flavus*	0.500	0.125	0.500	0.063	0.500	≤ 0.008	≤ 0.008
S005	F	52	Retreatment TB	*A. fumigatus*	0.250	0.125	0.250	0.063	0.500	≤ 0.008	≤ 0.008
				*A. flavus*	0.250	0.063	0.500	0.031	0.250	≤ 0.008	≤ 0.008
001	M	31	Post TB	*A. flavus*	0.250	0.125	1.000	0.063	0.500	≤ 0.008	≤ 0.008
18	M	24	Retreatment TB	*A. flavus*	0.500	0.125	0.500	0.031	0.500	≤ 0.008	≤ 0.008
004	F	58	Retreatment TB	*A. flavus*	0.500	0.063	0.250	0.031	0.500	≤ 0.008	≤ 0.008
08N	M	43	Retreatment TB	*A. fumigatus*	0.250	0.250	0.500	0.063	0.500	≤ 0.008	≤ 0.008
008N	M	41	Retreatment TB	*A. fumigatus*	0.250	0.125	0.250	0.063	0.500	≤ 0.008	≤ 0.008
				*A. flavus*	0.500	0.125	0.250	0.125	0.250	≤ 0.008	≤ 0.008
011N	F	38	Retreatment TB	*A. fumigatus*	0.250	0.125	0.250	0.031	0.500	≤ 0.008	≤ 0.008
13N	F	48	Retreatment TB	*A. flavus*	1.000	0.063	0.250	0.063	0.500	≤ 0.008	≤ 0.008
015N	F	48	Retreatment TB	*A. niger*	0.063	0.063	0.125	0.250	0.500	≤ 0.008	≤ 0.008
				*A. fumigatus*	1.000	0.250	1.000	0.063	0.500	≤ 0.008	≤ 0.008
				*A. flavus*	0.500	0.063	0.500	0.063	0.500	≤ 0.008	≤ 0.008
016N	M	40	Post TB	*A. flavus*	0.500	0.063	0.500	0.063	0.500	≤ 0.008	≤ 0.008

ID, identification; M, male; F, female; TB, tuberculosis.

### Antifungal susceptibility

Forty-seven *Aspergillus* isolates were tested using the CLSI M38-A microdilution method; anidulafungin and micafungin MICs were ≤ 0.008 µg/mL for all strains. For the *A. pseudonomiae* isolate, MICs were 0.5 µg/mL for amphotericin B, 0.25 µg/mL for itraconazole, 1 µg/mL for voriconazole, 0.125 µg/mL for posaconazole, and 1 µg/mL for isavuconazole. Using tentative breakpoints based on CLSI, no resistant isolates were found. Out of the four azoles, posaconazole showed the highest *in vitro* activity based on the lowest MIC_50_ of 0.063 µg/mL for *A. flavus, A. fumigatus* and *A. niger.* Itraconazole, voriconazole, and isavuconazole demonstrated the lowest *in vitro* activity, with a MIC_50_ value of 0.5 µg/mL against all three species. Notable MIC differences between species were observed for amphotericin B, with *A. flavus* having an MIC_50_ of ≥ 2 two-fold dilutions higher than *A. fumigatus* and *A. niger* (Table 2).

### Short tandem repeat genotyping

**FIGURE 2:**
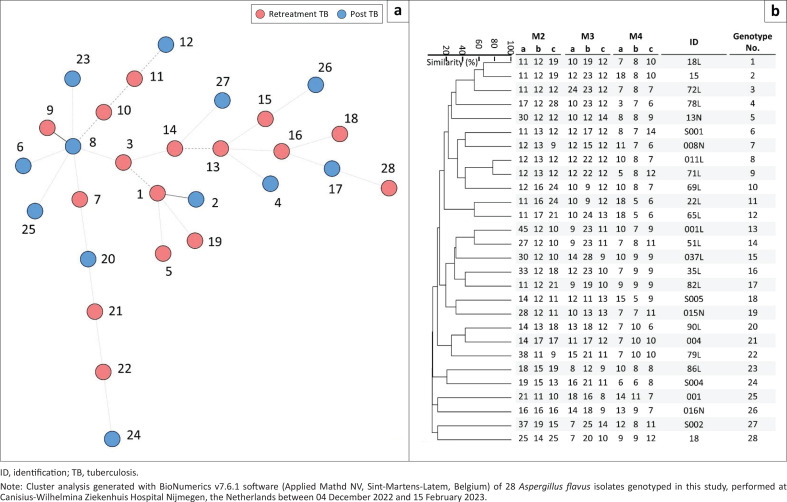
Cluster analysis of genotyped *Aspergillus flavus* isolates (*n* = 28) from Nigeria, between 01 June 2021 and 31 May 2022: (a) A minimum spanning tree with branch lengths indicating the similarity between isolates. Thin solid lines display variation in two markers, thin dashed lines variation in three markers and thin dotted lines in four or more markers (b) An Unweighted Pair-Group Method with Arithmetic Mean dendrogram displays copy numbers, isolate identifications (IDs), and genotype numbers.

**FIGURE 3:**
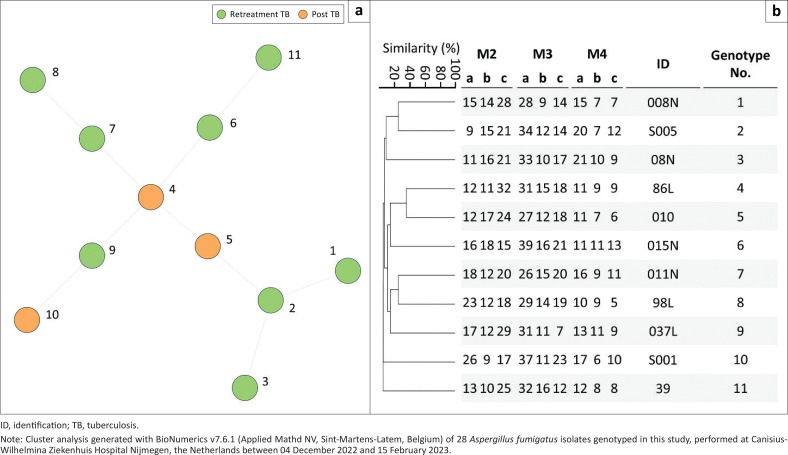
Cluster analysis of genotyped *Aspergillus* fumigatus isolates (*n* = 28) from Nigeria, between 01 June 2021 and 31 May 2022: (a) A minimum spanning tree with branch lengths indicating the similarity between isolates. Thin dotted lines display variation in four or more markers (b) An Unweighted Pair-Group Method with Arithmetic Mean dendrogram displays copy numbers, isolate identifications (IDs) and genotype numbers.

Using a multiplex polymerase chain reaction, nine markers in *A. fumigatus* and *A. flavus* strains were amplified, and then analysed to investigate the genotype relatedness with BioNumerics v7.6.1 software (Applied Mathd NV, Sint-Martens-Latem, Belgium). For both species, all isolates displayed unique genotypes (Figure 2 and Figure 3), and isolates did not cluster based on retreatment tuberculosis or post-tuberculosis treatment (Figure 2a and Figure 3a). All 28 *A. flavus* isolates differed from each other in at least three out of nine markers (Figure 2b), while *A. fumigatus* isolates differed in six or more short tandem repeat markers (Figure 3b).

## Discussion

This study demonstrated that CPA among the participants in this current study is more commonly associated with *A. flavus* than *A. fumigatus,* which is concordant with other studies from tropical countries. All clinical isolates were fully susceptible to azoles, echinocandins and amphotericin B. This is the first report of AFST in clinical isolates of *Aspergillus* in Nigeria.

The species distribution of *Aspergillus* isolates in this study (*A. flavus n* = 18, 35.3%; *A. fumigatus n* = 5, 9.8%; *A. niger, n* = 2, 3.9%) differs from studies conducted in Nigeria, where *A. fumigatus* recovered from sputum cultures of patients with HIV-tuberculosis co-infection accounted for 42.1% in the Northern part in 2019 and 57.14% in the Southwestern part in 2016.^28,29^ Cultures of 10 participants in the index study contained more than one *Aspergillus* spp., and it is not certain if these represented co-infections or merely colonisation. However, there were no significant differences in the *Aspergillus* spp. and the AFST between retreatment tuberculosis and post-tuberculosis patients. Only one *Aspergillus* isolate from a retreatment tuberculosis was identified as *A. pseudonomiae*. Phenotypic techniques can misidentify *Aspergillus* spp. with similar morphological features, and complementing with genotypic methods is expected to improve identification accuracy. Besides the common pathogenic *Aspergillus* spp., an isolate of *A. pseudonomiae*, which belongs to the section *Flavi*, was isolated. This isolate was initially identified as *A. fumigatus* based on colonial morphology and microscopic appearance. *Aspergillus pseudonomiae* was first isolated from soil and insects in the United States,^30^ and has only been isolated once so far from the environment in Nigeria; clinical infections are uncommon except for one report from a patient diagnosed with CPA.^31^ It is difficult to ascertain if the isolate from our study represents colonisation or was an actual cause of CPA, since only a single sputum specimen with *A. pseudonomiae* was collected from this patient who declined further participation. Besides this isolate, there was a high correlation between phenotypic identification and molecular methods in this study. While this may imply the reliability of phenotypic methods, especially for use in low-resource settings, the study’s relatively small sample size must be considered; therefore, further studies are needed before making definite recommendations.

Based on available clinical breakpoints and epidemiological cut-off values from the CLSI, all the *Aspergillus* strains in this study were susceptible to the tested antifungals, including azoles.^22,23^ Overall, the MICs for all the *Aspergillus* spp. were the same, except for amphotericin B against *A. flavus.* Several other studies have reported higher amphotericin B MICs against *A. flavus* when compared to other *Aspergillus* spp.^31^ It is thought that *A. flavus* displays higher ergosterol levels with increased activity of peroxidase and superoxide dismutase, resulting in reduced susceptibility.^32^ Since high MICs to amphotericin B are associated with therapeutic failure, voriconazole is the preferred drug for treating all forms of invasive aspergillosis.^31^

The absence of triazole resistance in *Aspergillus* spp. in this study was not surprising, because the study participants had no prior exposure to azole drugs and no reports of environmental or cross-resistance to triazoles have been reported in the region to date; a study conducted on soil samples in Lagos Nigeria reported that all the *Aspergillus* spp. isolated were susceptible to voriconazole and itraconazole.^33^ In contrast, another study reported 2.0 to 2.2% prevalence from soil samples collected in West Africa, including Burkina Faso and Nigeria.^14^ However, the study site of the reported azole resistance *Aspergillus* spp. in Nigeria was outside Lagos. In East Africa, triazole-resistant *Aspergillus* strains have been reported, from agricultural settings and clinical isolates in Tanzania and Kenya.^15,34,35,36^ Outside Africa, the prevalence of azole-resistant clinical isolates in Europe steadily increased from 1.7% in 1997 to 9% in 2009,^37^ and 15% as of 2018,^38^ while it was 4.3% in a single centre study from China,^39^ and 3.5% in the United States.^40^ The exponential increase in agricultural azole fungicides used for crop protection and material preservation against phytopathogens may be responsible for observed rates of azole-resistant *Aspergillus* spp. in these regions.^10,41,42^ Meanwhile, information about azole fungicides in Africa is scarce and only the study conducted in Kenya described azole resistance from azole-naïve and azole-experienced soil samples.^15^ Thus, the lack of surveillance studies may have resulted in the low prevalence reported in Africa.^14^ Therefore, coordinated data collection with a standardised protocol and continuous surveillance of environmental and clinical *Aspergillus* spp. is warranted in Nigeria.

The absence of *A. fumigatus* and *A. flavus* isolates with identical or highly related short tandem repeat genotypes suggests no transmission of these fungal species between patients within or between hospitals. For *A. fumigatus,* microsatellite typing has extensively been applied and suggests a high genetic diversity.^28,40^

### Limitations

The results of this study may not be generalisable to other locations within Nigeria. Other limitations include the relatively small number of isolates and the high frequency of mixed cultures, as it was not possible to determine if these represented co-infections or colonisation. The limitations notwithstanding, the data generated support using azoles as first-line therapy for CPA treatment in the study location.

### Conclusion

Molecular species identification helped to identify the *Aspergillus* strains accurately. Antifungal susceptibility results showed susceptible strains in this study, suggesting that de novo or environmentally mediated resistance is unlikely to be an issue in managing CPA patients. However, given the long-term treatment required, surveillance for resistance development during therapy is warranted. Systematic surveillance of clinical and environmental *Aspergillus* spp. is needed in Nigeria to monitor for the emergence of azole-resistant phenotypes.
